# Identification of genotype 4 Hepatitis E virus binding proteins on swine liver cells

**DOI:** 10.1186/1743-422X-8-482

**Published:** 2011-10-27

**Authors:** Wen Zhang, Xiuguo Hua, Quan Shen, Shixing Yang, Hong Yin, Li Cui

**Affiliations:** 1 School of Agriculture and Biology, Shanghai JiaoTong University, 800 Dongchuan Road, Shanghai 200240, China; 2 School of Medical Science and Laboratory Medicine, Jiangsu University, 301 Xuefu Road, Zhenjiang, Jiangsu 212013, China; 3 State Key Laboratory of Veterinary Etiological Biology, Lanzhou Veterinary Research Institute of Chinese Academy of Agricultural Sciences, Lanzhou, Gansu 730046, China

**Keywords:** Hepatitis E virus, Receptor, VOPBA, Mass Spectrometry Fingerprinting

## Abstract

Hepatitis E virus (HEV) is a zoonotic pathogen of which several species of animal were reported as reservoirs. Swine stands out as the major reservoir for HEV infection in humans, as suggested by the close genetic relationship of swine and human virus and cross-species infection of HEV. Up to now, the mechanism of cross-species infection of HEV from swine to humans is still unclear. This study sought to identify receptor element for genotype 4 HEV on swine liver cells using the viral overlay protein binding assay (VOPBA) technique and Mass Spectrometry fingerprinting. A single virus binding band with natural molecular weight about 55 kDa was observed, and mass spectrometry revealed that this virus binding band contained 31 different proteins. Infection inhibition assay suggested that this 55 kDa protein could prevent HEV from infecting its susceptible A549 cell line, which was further confirmed by the HEV genome detecting in the inoculated cells. Further research should be performed to elucidate the accurate receptor of HEV on the swine liver cells.

## Introduction

Hepatitis E virus (HEV), a member of the genus Hepevirus, is a non-enveloped virus with a positive-stranded RNA genome approximately 7.2 kb in length, which consists three open reading frames (ORF1-3)[1]. ORF1 locates at the 5' of genome and encodes non-structural proteins, including the methyltransferase, protease, helicase and RNA-dependent RNA polymerase (RdRp) [2]. ORF2 maps to the 3' terminus and encodes for a major structural protein, and ORF3 overlaps both and encodes a thus far unknown function [3]. It has been hypothesised that zoonosis is involved in the transmission of HEV [4]. HEV isolates were divided into 4 distinct genotypes which were recently proposed to be further classified into 24 subtypes [5]. Genotypes 1 and 2 have been identified exclusively in humans, while genotypes 3 and 4 have been found in humans and several species of animals. Swine stands out as a reservoir for hepatitis E virus (HEV) infection in humans, as suggested by the close genetic relationship of swine and human virus and cross-species infection of HEV [6,7].

Viral overlay protein binding assay (VOPBA) has been used to identify receptor proteins for various viruses, such as Bovine adenovirus [8], Dengue Virus [9], Fowl Adenovirus [10], and pancreatic necrosis virus [11]. This assay is sensitive enough to detect the binding of less than 100 mg of protein in a crude membrane preparation or to detect 5 mg of purified glycophorin [12]. As a cross-species infection pathogen, identification of receptor of HEV to enter cells is very important for elucidating the mechanisms of cross-species infection between humans and animals. However, there is little information on the interaction between HEV and liver cells nowadays, therefore, the present study aimed to use VOPBA combining MS analysis to identify the virus binding protein on the swine liver cell.

## Materials and Methods

### Viruses Sample and Purification of Viral Particles

HEV positive swine fecal sample was form an experimentally infected pig with a genotype 4 HEV strain (GenBank accession no.: EF570133). This sample was proved to be negative for PEVs (including PTV and PEV1-10), haemagglutinating encephalomyelitis virus, Aujeszky's disease virus, porcine circovirus type 2, porcine reproductive and respiratory syndrome virus, classical swine fever virus, Japanese encephalitis virus, porcine transmissible gastroenteritis virus, porcine epidemic diarrhoea virus, porcine rotavirus, porcine sapovirus, cytomegalovirus, porcine Torque-Teno virus and porcine parvovirus by RT-PCR/PCR methods. The fecal sample was converted to 10% (w/v) suspensions in PBS (pH7.4) and clarified by centrifugation at 15, 000 g for 30 min. Supernatants were purified by passage through 0.22 μm microfilters (Millex-GV, Millipore) before virus inoculation or purification for viral overlay protein binding assay (VOPBA). For purification of viral particles, 150 ml of the purified virus suspension were mixed with polyethylene glycol 6000 (PEG 6000) and NaCl to final concentrations of 6% and 0.3 M, respectively. The mixture was stirred at 4°C for 6 h, then centrifuged at 20, 000 g for 90 min at 4°C. The precipitated sediment was resuspended in 15 ml PBS. A 10-60% (w/v) discontinuous sucrose density gradient was made in a Beckman 25 × 76 mm Ultraclear tube (Beckman Instruments, USA) using a modification of the method described previously [13]to purified the HEV particles. The sample between 20% and 30% was collected and proved to contain HEV particles, of which the virus antigens were detected by western blotting [14,15] and virus particles were observed by Electron Microscope (EM) [16].

### Membrane Protein Preparation

Swine liver tissue was gifted from the Institute Animal Medicine of Shanghai Jiaotong University. Tissues were first ground with Liquid Nitrogen in mortar and membrane protein was extracted from swine liver tissue using Plasma Membrane Protein Extraction Kit (BioVision, USA) in accordance with the manufacturer's protocol. The concentration of protein was quantified by the Bradford method [17].

### Viral Overlay Pprotein Binding Assay (VOPBA)

Membrane proteins (60 to 100 μg) were subjected to electrophoresis through an 8% sodium dodecyl sulfate (SDS)-polyacrylamide gel and transferred to PVDF membrane (Millipore, Billerica, MA, USA). The membrane containing transferred proteins was blocked with 5% skim milk in TBS at room temperature for 1 h. The membranes were incubated with suitable amount of purified HEV particles in 1% skim milk in TBS for 12 h at room temperature and washed three times with TBS buffer. Subsequently, the membranes were incubated with a specific rabbit anit-HEV-ORF2 antibody (Affinity BioReagents, USA) at a dilution of 1:100 in 5% skim milk in TBS buffer. The viral binding band was visualized by incubation with a secondary horseradish peroxidase-conjugated sheep anti-rabbit IgG. Finally, the signal was developed using the ECL Advanced™ Western Blotting Detection kit (GE Healthcare Bio-Sciences, UK) in accordance with the manufacturer's protocol.

### Mass Spectrometry

Mass spectrometry was undertaken commercially by the Research Centre for Proteome Analysis, Shanghai Institutes for Biological Sciences, Chinese Academy of Sciences, Shanghai, China. Band was subjected to tryptic digestion for 16 h followed by Matrix Assisted Laser Desorption Ionization (MALDI) mass spectrometry and was performed with a Micromass Maldi Time of Flight (MALDI-TOF) Mass Spectrometer. Spectra were acquired in the mass range 400-2000 Da. Spectra were searched against Suina Protein Data Bank in NCBI.

### Inhibition of Infection

Proteins in band were electroeluted using D-Tube Dialyzers & D-Tube Electroelution Kit (Novagen, Darmstadt, Germany) in accordance with the manufacturer's protocol. The purified HEV supernatant was pre-incubated with eluted proteins, or purified swine fecal suspension which was collected from SPF pigs, and was then incubated with the human lung carcinoma A549 cell line according to the previous report [18]. The cytopathic effects (CPE) were then investigated and HEV genomes in infected cells were detected by RT-PCR method [19].

## Results

### Virus Particles and Antigens

**Figure 1:**
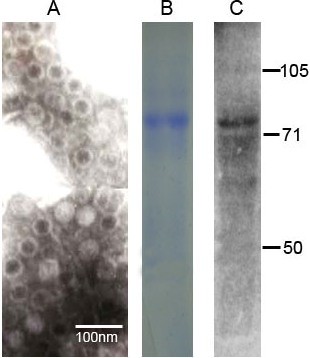
**Identification of virus particles and antigens in the sucrose density gradient centrifugation purified sample**. (A)The transmission electron micrographs show the HEV particles with a diameter of 30 nm; (B) and (C) the SDS-PAGE and western blotting results of the purified samples, respectively, where we can see the 75kDa bands of HEV capsid proteins. Position of protein marker bands are shown in kDa

To confirm the existence of the HEV particles and antigens, the sucrose density gradient centrifugation purified sample were subjected to EM and western blotting observation. Results were presented in Figure 1. Figure 1A indicated the transmission electron micrographs of HEV particles in the purified sample, with a diameter about 30 nm. Figure 1B and 1C showed the SDS-PAGE and western blotting results of the purified samples, respectively, where we can see the bands of HEV capsid proteins with molecular weight about 75kDa. These results proved that the purified sample contained applicative HEV particles which could be used for the VOPBA method.

### Viral Overlay Protein Binding Assay

**Figure 2:**
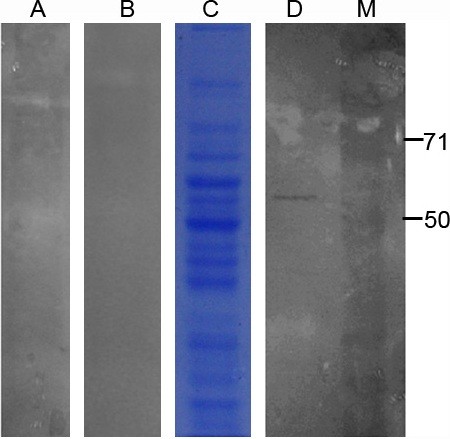
**VOPBA analysis of HEV on swine liver cell membrane proteins**. (A)The electroeluted protein was pre-incubated with virus particles before hybridization; (B) the transferred protein was directly incubated with the anit-HEV-ORF2 antibody without incubating with virus particles; (C) gel containing the swine liver cell membrane proteins was stained with Coomassie brilliant blue; (D) The PVDF membrane was incubated with virus particles and western blotting was then performed using a specific rabbit anit-HEV-ORF2 antibody, a single virus binding band about 55kDa was observed. Position of protein marker bands are shown in kDa

To preliminarily identify the molecules on swine liver cells involved in binding HEVs, the virus overlay protein binding assay (VOPBA) methodology was utilized. Membrane proteins from swine liver cells were isolated and separated by SDS-PAGE on two parallel 8% gels. One gel was stained with Coomassie brilliant blue R-250 (Figure 2C), while the other gel was transferred to PVDF membrane by wet electroblotting. The resulting PVDF membrane was hybridized with HEV particles. A single virus binding band of approximately 55 kDa was visualized by subsequent western blotting using a specific rabbit anit-HEV-ORF2 antibody (Figure 2D). To investigate the band was not directly produced by the anit-HEV-ORF2 antibody with the proteins on the PVDF membrane, one separate PVDF membrane with transferred protein was directly incubated with the anit-HEV-ORF2 antibody without incubating with virus particles. No specific binding band was visualized (Figure 2B), which suggested the 55 kDa binding band was produced by the interaction between HEV particles and the transferred proteins. To investigate the position in the gel stained with Coomassie brilliant blue was corresponding to the position of the VOPBA binding band, the position equivalent to the major virus binding band was extracted from the gel and the proteins in the gel was electroeluted. The electroeluted protein was then pre-incubated with virus particles before hybridization between virus particles and the PVDF membrane containing transferred membrane proteins. Result was indicated in Figure 2A, where there was no specific binding band. This result suggested that corresponding position of the gel could be used for the subsequent inhibiting assay and mass spectrometry fingerprint analysis.

### Mass Spectrometry Fingerprint Analysis

**Figure 3:**
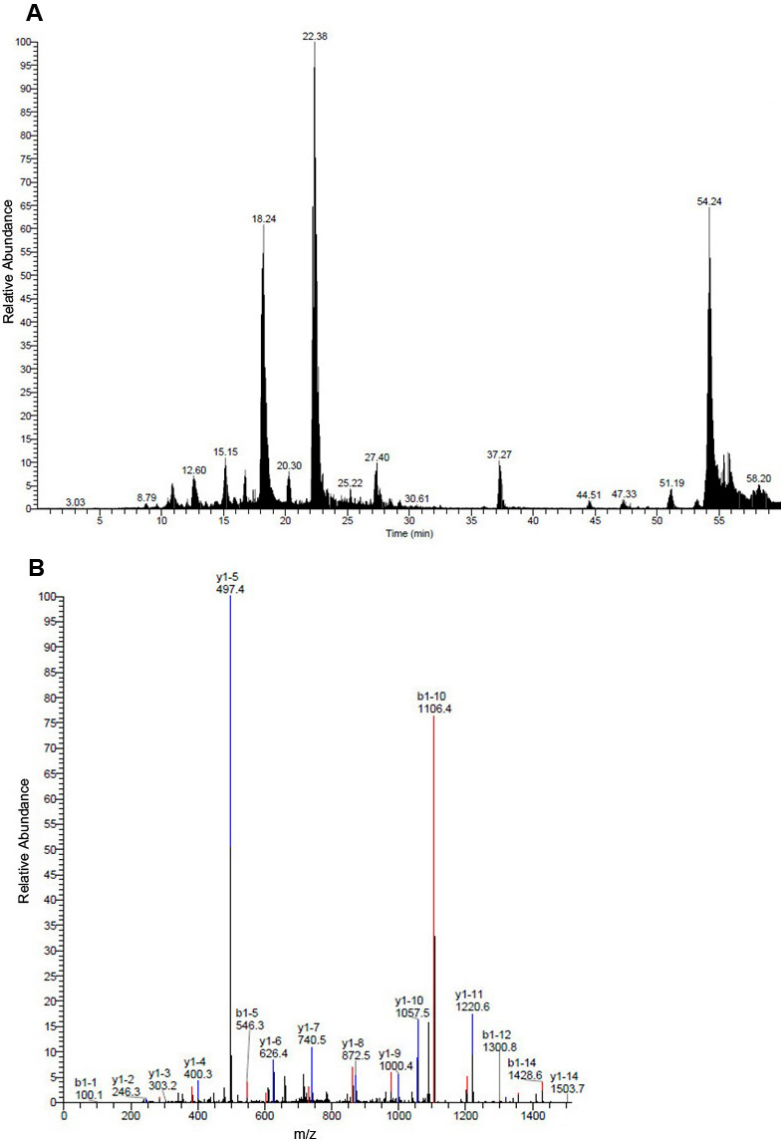
**Basepeak (A) and peptide mass fingerprinting (B) pictures of MALDI-TOF analysis of dengue virus binding band from VOPBA experiment**.

To investigate the protein content of the virus binding band, the position equivalent to the major virus binding band was extracted from the duplicate gel and sent for commercial mass spectrometry fingerprint analysis. The basepeak and peptide mass fingerprinting of MALDI-TOF analysis of HEV binding band from VOPBA experiment were shown in Figure 3A and 3B, respectively. Protein search results indicated that proteins in the virus binding band was rather complicated, containing 31 different proteins (Additional file 1). These results meant that the accurate binding protein couldn't be identified based on the mass spectrometry analysis.

### Inhibition of Virus Infection

**Figure 4:**
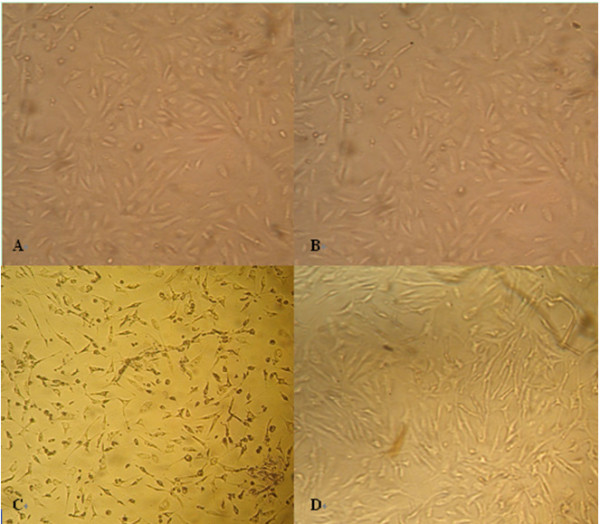
**Microscope of A549 cell lines seven days post-inoculation**. (A) cells inoculated with SPF pig fecal suspension, without evident CPE; (B) cells inoculated only with electroeluted virus binding proteins, without evident changes; (C) cells inoculated with virus suspension, there was evident CPE; (D) cells inoculated with the mixture of virus suspension and electroeluted virus binding proteins, there were little CPE, which were significantly less than that in (C).

To investigate whether the electroeluted protein from the virus binding band could inhibit the HEV infection of the susceptible cell line A549, an infection inhibiting assay was performed. Before infection, HEV suspension was pre-incubated with the electroeluted protein, and then the mixture was used to infect the A549 cell line. The cell shape was investigated every 24 hours and the 7^th ^day results were indicated in Figure 4. Figure 4A showed the cells in negative control, which were inoculated with SPF pig fecal suspension, testing the effect of the fecal suspension without any pathogen on A549 cell line. There was no evident CPE in this control group, which suggested that the fecal suspension had no effect on the A549 cell line. Figure 4B showed the results of cells that were inoculated only with electroeluted virus binding proteins for testing the effect of the virus binding proteins on A549 cell lines. There was no evident changes in this group, which suggested that the electroeluted virus binding proteins had no significant effect on the cell line. Figure 4C indicated the cells that were infected by virus suspension, where there was evident CPE. Figure 4D indicated the infection inhibiting group, where the mixture of virus suspension and electroeluted virus binding proteins were used to inoculated to A549 cells. In this group, there were little CPE phenomena, which were significantly less than that in Figure 4C, which suggested that the virus binding protein could inhibit the infection of HEV to the susceptible A549 cell line. In order to investigate whether HEV genome could be detected in these groups, RT-PCR method was used, and results indicated that only the cells shown in Figure 4C were positive for HEV genome, which confirmed that the virus binding protein could inhibit the infection of HEV to the susceptible A549 cell line.

## Discussion

There is gathering evidence that HEV is enzootic and pigs are considered one of the major reservoirs of human infection with this virus. The zoonotic transmission of HEV from pigs to humans has been suggested previously, particularly for cases in non-endemic areas. This hypothesis was mainly based on phylogenetic analysis, which showed that swine and human HEV strains from same geographic regions share a high genomic similarity [4,7,20-22]. The initial interaction between a cell and a virus is a critical determinant of viral tropism and thus of pathogenicity; therefore, considerable interest lies in determining the nature of the proteins used by viruses to enter cells. While infections with HEV present a significant worldwide problem, especially the regions with poor sanitation. Although some research about the putative receptor-binding sites on HEV capsid protein has been performed [23], little progress has been made in identifying the extracellular proteins utilized by HEV to gain entry into cells. Therefore, identification of receptor of HEV to enter cells is very important for elucidating the mechanisms of cross-species infection between humans and animals and for the development of gene-engineering vaccine.

Using a combination of VOPBA and MS analysis, we have identified a virus binding protein on the swine liver cell with nature molecular weight about 55kDa. The infection inhibition analysis indicated that this virus binding protein could prevent the HEV from infecting the A549 cell line [Figure 4]. The peptide fingerprint analysis and protein search showed that the virus binding band containing 31 different proteins (Additional file 1). This meant that the accurate receptor of HEV couldn't be identified in the present study. However, among the 31 proteins, four proteins could be ever identified as receptor of virus or other proteins. They are Integral membrane protein 2B [24,25], Moesin-B [26], ATP synthase subunit alpha liver isoform [27], and Cytochromo P450 [28]. Next step, we will aim to clone these genes form pigs and humans to further elucidate whether they can be used as HEV receptors.

In the present study, we only used genotype 4 HEV for identifying the virus binding proteins in swine liver cells, because among the four genotypes of HEV, only genotype 3 and 4 belong to zoonotic pathogens. This study will provide some reference for the research of identifying the binding proteins of the other HEV genotypes. Although the aim of our study was identifying the binding proteins on the surface of swine cell so as to finding the candidate receptor of HEV, some of proteins in the aim band were not membrane proteins, which may be due to slight solubility of membrane proteins. Therefore, more effective method for membrane protein extraction should used during the future research for identifying the receptors of virus using VOPBA methods.

## Conclusion

Taken together, we have identified a virus binding protein on the swine liver cell with nature molecular weight about 55kDa by using a combination of VOPBA and MS analysis, and this virus binding protein could prevent the HEV from infecting the A549 cell line. The peptide fingerprint analysis and protein search showed that this virus binding band containing 31 different proteins. Although the accurate receptor of HEV has not been identified, the present study will lay a foundation for further research of the receptor of HEV.

## Competing interests

The authors declare that they have no competing interests.

## Authors' contributions

WZ, LC, and XH conceived the study. WZ, QS, SY, and HY performed all the experiments. WZ and LC wrote the paper. All authors read and approved the final manuscript.

## Supplementary Material

Additional file 1 **Protein component of the HEV binding band from VOPBA**. The protein band was subjected to MALDI-TOF and the resulted mass spectra were searched against Suina Protein Data Bank in NCBI. The table listed the proteins in the binding band which was rather complicated and contained 31 different proteins. Click here for file
