# Supplementary material for: Identification of genotype 4 Hepatitis E virus binding proteins on swine liver cells
Source: Virol J. 2011 Oct 27;8:482. doi: 10.1186/1743-422X-8-482 (PMC3219747; doi:10.1186/1743-422X-8-482)
Supplement: Additional file 1 — Protein component of the HEV binding band from VOPBA. The protein band was subjected to MALDI-TOF and the resulted mass spectra were searched against Suina Protein Data Bank in NCBI. The table listed the proteins in the binding band which was rather complicated and contained 31 different proteins. [file 1743-422X-8-482-S1.DOC]

| Group number | Protein name |
| --- | --- |
| 1 | mitochondrial ATP synthase, H+ transporting F1 complex beta subunit |
| 2 | Epoxide hydrolase 1 |
| 3 | Serum albumin precursor |
| 4 | Elongation factor 1-gamma |
| 5 | oligosaccharyltransferase OST48 |
| 6 | Chain A, The High Resolution Crystal Structure Of Porcine Pepsinogen |
| 7 | Beta-enolase (2-phospho-D-glycerate hydro-lyase) |
| 8 | eukaryotic translation elongation factor 1 alpha 1 |
| 9 | Rab GDP dissociation inhibitor beta (Rab GDI beta) |
| 10 | Potassium-transporting ATPase subunit beta |
| 11 | serine (or cysteine) proteinase inhibitor, clade F, member 1 |
| 12 | ribosomal protein L4 |
| 13 | Spliceosome RNA helicase BAT1 |
| 14 | Chain A, Crystal Structure Of Mitochondrial Respiratory Complex Ii |
| 15 | Endoplasmin precursor (Heat shock protein 90 kDa beta member 1) |
| 16 | cathepsin D |
| 17 | Actin, cytoplasmic 1 (Beta-actin) |
| 18 | Chain A, The Crystal Structure Of Diferric Porcine Serum Transferrin |
| 19 | Alpha-2-HS-glycoprotein precursor (Fetuin-A) |
| 20 | Chain A, Crystal Structure Of Porcine Beta Trypsin |
| 21 | Integral membrane protein 2B |
| 22 | Radixin (Moesin-B) |
| 23 | actin-related protein 3 |
| 24 | cytochrome P450 2C49 |
| 25 | interleukin 6 signal transducer |
| 26 | acyl-CoA synthetase long-chain family member 4 |
| 27 | immunoglobulin heavy chain variable region |
| 28 | IgG heavy chain |
| 29 | Dihydrolipoyllysine-residue succinyltransferase component of 2-oxoglutarate dehydrogenase complex, mitochondrial precursor |
| 30 | Sodium/potassium-transporting ATPase subunit beta-1 |
| 31 | ATP synthase subunit alpha liver isoform |

Supplementary table: The 31 different proteins contained in the virus binding band
